# OVER 1 MILLION TREATED WITH HIGHLY EFFECTIVE HEPATITIS C MEDICINES

**Published:** 2017-01

**2 SEPTEMBER 2016 | GENEVA** - 27 OCTOBER 2016 | GENEVA - Over one million people in low- and middle-income countries have been treated with a revolutionary new cure for hepatitis C since its introduction two years ago.

When Direct Acting Antivirals (DAAs) were first approved for hepatitis C treatment in 2013, there were widespread fears that their high price would put them out of reach for the more than 80 million people with chronic hepatitis C infections worldwide.

The new medicines have a cure rate of over 95%, fewer side effects than previously available therapies, and can completely cure the disease within three months. But at an initial estimated price of some US$85 000 they were unaffordable even in high-income countries.

**Countries show hepatitis C treatment is achievable**

Thanks to a series of access strategies supported by the World Health Organization (WHO) and other partners, a range of low- and middle-income countries, including Argentina, Brazil, Egypt, Georgia, Indonesia, Morocco, Nigeria, Pakistan, Philippines, Romania, Rwanda, Thailand and Ukraine – are beginning to succeed in getting drugs to people who need them. Strategies include competition from generic medicines through licensing agreements, local production and price negotiations.

“Maximizing access to lifesaving hepatitis C treatment is a priority for WHO,” says Dr Gottfried Hirnschall, Director of WHO’s Department of HIV and Global Hepatitis Programme. “It is encouraging to see countries starting to make important progress. However, access still remains beyond the reach for most people.”

A new WHO report, Global Report on Access to Hepatitis C Treatment: Focus on Overcoming Barriers, released today shows how political will, civil society advocacy and pricing negotiations are helping address hepatitis C, a disease which kills almost 700 000 people annually and places a heavy burden on health systems’ capacities and resources.

“Licensing agreements and local production in some countries have gone a long way to make these treatments more affordable,” says Dr Suzanne Hill, WHO Director for Essential Medicines and Health Products. For example, the price of a three-month treatment in Egypt dropped from US$900 in 2014 to less than US$200 in 2016.

“But there are still huge differences between what countries are paying. Some middle-income countries, which bear the largest burden of hepatitis C, are still paying very high prices. WHO is working on new pricing models for these, and other expensive medicines, in order to increase access to all essential medicines in all countries,” says Dr Hill.

**80% of people in need still face challenges**

Among middle-income countries, the price for a three-month treatment of sofosbuvir and daclatasvir varies greatly. Costs range from US$9 400 in Brazil to US$79 900 in Romania.

High costs have led to treatment rationing in some countries, including in the European Union, where price agreements have not accounted for the full cost of treating the whole affected population.

“Today’s report on access, prices, patents and registration of hepatitis C medicines will help create the much needed market transparency which should support country efforts to increase access to DAAs,” said Dr Hirnschall. “We hope countries will update their hepatitis treatment guidelines, work to remove barriers to access, and make these medicines available promptly for everyone in need.”

In May 2016, at the World Health Assembly, 194 countries adopted the first-ever Global Health Sector Strategy on Viral Hepatitis, agreeing to eliminate hepatitis as a public health threat by 2030. The strategy includes a target to treat 80% of people in need by this date.

WHO issued guidelines recommending the use of DAAs in 2014 and 2016 and included DAAs on its Essential Medicines List – which is compiled to address the priority healthcare needs of populations; to make needed essential medicines available at all times in adequate amounts, at a price the health system and community can afford.

Available from: http://www.who.int/mediacentre/news/releases/2016/hepatitis-c-medicines/en/

